# A multidisciplinary non-invasive approach to monitor response to intravenous immunoglobulin treatment in neurodegenerative Langerhans cell histiocytosis: a real-world study

**DOI:** 10.3389/fimmu.2024.1422802

**Published:** 2024-08-16

**Authors:** Irene Trambusti, Carmen Barba, Marzia Mortilla, Susanna Rizzi, Katiuscia Romano, Maria Luisa Coniglio, Ersilia Lucenteforte, Annalisa Tondo, Renzo Guerrini, Elena Sieni

**Affiliations:** ^1^ Pediatric Hematology-Oncology Department, Meyer Children’s Hospital IRCCS, Florence, Italy; ^2^ Neuroscience and Human Genetics Department, Meyer Children’s Hospital IRCCS, Florence, Italy; ^3^ University of Florence, Florence, Italy; ^4^ Pediatric Radiology Department, Meyer Children’s Hospital IRCCS, Florence, Italy; ^5^ Department of Statistics, Computer Science and Applications “G. Parenti”, University of Florence, Florence, Italy

**Keywords:** neurodegenerative Langerhans cell histiocytosis, evoked potentials, immunoglobulin, SEPs, ND-LCH

## Abstract

**Aims:**

Early detection and treatment of neurodegenerative Langerhans cell histiocytosis (ND-LCH) have been suggested to prevent neurodegenerative progression. The aim of the study is to validate a standardized multidisciplinary diagnostic work-up to monitor the intravenous immunoglobulins (IVIG) treatment response and the natural course of the disease in untreated patients.

**Methods:**

Patients with abnormal somatosensory evoked potentials (SEPs) received monthly 0.5 g/kg IVIG. The diagnostic protocol included structural 3T MRI, neurological examination, brainstem auditory evoked potentials (BAEPs) and SEPs.

**Results:**

Twenty-two patients were followed for 5.2 years (median) from the first MRI evidence of ND-LCH. Eleven patients received IVIG for 1.7 years (median). At treatment start neurological examination was abnormal in 10 patients, of whom two had severe clinical impairment and four had abnormal BAEPs. At last follow-up, 1/11 remained stable and 7/11 improved, while worsening of neurological or neurophysiological findings, or both, occurred in 3/11. Risk factors for worsening were a severe clinical or MRI ND-LCH at treatment initiation and prolonged exposure to LCH. Of the 11 untreated patients, none improved and three worsened.

**Conclusions:**

Using a standardized diagnostic protocol, we demonstrated that IVIG treatment can lead to clinical stabilization or improvement in all pauci-symptomatic patients with an MRI grading of less than 4.

## Background

Neurodegenerative Langerhans cell histiocytosis (ND-LCH) is a potentially devastating, late-onset and progressive consequence of Langerhans cell histiocytosis (LCH). It is characterized by a typical MRI pattern of bilateral and symmetric T2 and FLAIR hyperintense lesions in the cerebellar grey matter, sometimes extending to the underlying white matter, in the basal ganglia and brainstem (1–3). Its prevalence ranges between 4% and 24% in different cohorts of children and young adults with LCH (3–5). Clinical ND-LCH develops after a median of 4.2 years from LCH diagnosis (6). Its 15-year cumulative incidence has been estimated to be between 1.8% and 8.6% in the French LCH registry (7). Underlying pathogenic mechanisms ND-LCH remain to be elucidated. Based on histopathological findings, ND-LCH appears to be mediated by CD8-positive lymphocytes and neuroinflammatory cytokines/chemokines directly causing tissue damage or initiating an autoimmune response to brain components (8). A possible role of the extracellular-signal-regulated kinase (ERK) pathway in ND-LCH has been suggested (9), since neurodegeneration can be driven by a somatic mutation occurring in the yolk sac erythromyeloid progenitors in a mouse model. Alternatively, the finding of *BRAF* ^V600E+^ cells in peripheral blood mononuclear cells and infiltrating myeloid/monocytic cells in the brain of patients with ND-LCH might suggest a mutated hematopoietic precursor (10). Indeed, bone morrow-derived CD11a+ macrophages infiltrating the brain have been recently suggested to contribute to ND-LCH pathogenesis in mouses (11).

Patients with diabetes insipidus (DI) secondary to pituitary lesions (12, 13) and/or craniofacial bone lesions (14) are particularly at risk to develop ND-LCH. Recently, lesional *BRAF* ^V600E+^ status was suggested as an additional risk factor (7).

Despite a relatively homogeneous MRI pattern, the clinical picture of ND-LCH is very heterogeneous, ranging from mild to severe neurological impairment, including tremor, abnormal osteotendinous reflexes, spasticity, gait disturbance up to severe ataxia, cognitive and psychiatric symptoms (3, 4, 15–18). Not all patients with imaging findings suggestive of ND-LCH develop clinical symptoms, even many years after the initial diagnosis (3).Despite all efforts made over the last 20 years to better understand epidemiology, risk factors and pathogenesis of ND-LCH, an effective treatment is still lacking. Several approaches, including retinoic acid (19), chemotherapy i.e., the combination of vincristine and cytarabine (20), and intravenous immunoglobulins (IVIG) (21), were used in clearly symptomatic ND-LCH patients, thus obtaining, at best, a stabilization of the neurological symptoms. The use of IVIG in ND-LCH has been proposed due to immune-modulatory effects in other neuro-inflammatory disorders such as multiple sclerosis (22). Recently, targeted therapies with mitogen-activated protein kinase (MAPK) inhibitors have shown efficacy in a ND-LCH mouse model induced by *BRAF* ^V600E^ expression in microglia (9) and in a few patients (10, 23), especially after early treatment initiation. However, the lack of standardized diagnostic evaluations prevents clinicians from drawing firm conclusions only based on the studies mentioned above.

Owing to these diagnostic uncertainties, a standardized diagnostic approach able to monitor treatment efficacy in pauci-symptomatic or asymptomatic patients represents a response to an unmet medical need. We had previously proposed a multidisciplinary protocol including 3T brain MRI, neurological and neurophysiological assessment i.e., brainstem auditory evoked potentials (BAEPs) and somatosensory evoked potentials (SEPs) for detecting the early stages of ND-LCH. We observed that while structural MRI represents the reference standard for diagnosis, SEPs have the highest capability to predict ND-LCH and discriminate its grading (6). Based on our preliminary data, the North American Consortium for Histiocytosis has proposed evoked potentials as complementary to neurological and neuroimaging assessments, for clinical evaluation and monitoring of patients with ND-LCH (level of Evidence 3iii) (24). Since 2015, SEPs have been increasingly used in our center as well as in others throughout Italy for the early selection of patients with ND-LCH to be submitted to IVIG treatment (25). The choice of IVIG at the time of planning this study was motivated by the well tolerated profile and the promising results of a previous Japanese study showing stabilization of neurologic symptoms in four of five patients treated with monthly IVIG (in combination with chemotherapy for concomitant active disease in 3 of them) for a median time of 23 months (26). This finding was confirmed by a follow-up study of 8 patients treated with IVIG for a median period of 6.5 years (21). More recently, cerebrospinal fluid (CSF) biomarkers including osteopontin (10) and neurofilament light (27) have been reported in association with ND-LCH. Neurofilament light appears promising in monitoring the response of MAPK inhibitors in a pilot study including 4 patients with ND-LCH, but it requires a lumbar puncture and sedation in children (27). Conversely, plasma neurofilament light does not correlate well with CSF levels and its utility as a screening test for ND-LCH is currently under evaluation (28).

The aim of the present study is to prospectively validate a standardized, non-invasive, multi-disciplinary diagnostic protocol for the monitoring of ND-LCH patients undergoing IVIG treatment. We also sought to identify early predictors of non-response to IVIG. Additionally, we aimed to enhance the understanding of the natural course of untreated ND-LCH.

## Methods

### Objectives

To follow-up patients with ND-LCH using the multidisciplinary standardized diagnostic protocol we proposed in a previous study (6) to monitor:

1. the response to IVIG therapy in pauci-symptomatic   ND-LCH patients.

2. the natural history of untreated patients with ND-LCH.

### Study population and protocol

Since 2010 a prospective observational study of patients with LCH and risk factors for ND-LCH has been ongoing at Meyer Children’s University Hospital in Florence, Italy, which serves as the national referral center for histiocytosis.

In line with our previous study (6), all patients with risk factors for ND-LCH were evaluated using a diagnostic protocol including: 3T structural MRI and MR spectroscopy (MRS), neurological examination (NE), including the Scale for ataxia (SARA), and neurophysiology (SEPs, BAEPs). For details see Sieni et al. **Supplementary Materials** (6).

Patients diagnosed with ND-LCH were subsequently followed-up yearly using the same multidisciplinary diagnostic protocol.

From April 2017 to December 2021 a therapeutic study was conducted with the following inclusion criteria: typical MRI findings of ND-LCH and at least SEPs alterations. Therapy consisted of monthly administrations of 0.5g/kg IVIG according to previous studies (21, 26). The treatment response was evaluated annually with the same diagnostic protocol. Patients were considered as “treated” if they received at least one year of IVIG and were available for follow-up assessments.

At follow-up assessments, “treated” patients with stable or improved results in at least one examination (neurological or neurophysiological) were considered “stable” and compared with patients who worsened in at least one of the above-mentioned parameters.

The MRI severity was assessed by a previously reported cerebellar grading (6) scored 1 to 4 to indicate the grade of cerebellar lesion involvement from mild to very severe. The severity of grading was assessed either by the extension of the lesions and their signal intensity.

A neurological examination was considered as worsened if 1) new neurological signs or symptoms had appeared 2) the SARA score had increased. BAEPs were considered as worsened if a) they became abnormal i.e., low amplitude, delayed latency, or absence of III or IV potentials according to reference values, or b) there was further worsening of already abnormal responses. SEPs were deemed as worsened if worsening of at least one response could be demonstrated (latency, amplitude).

Patients without treatment indication were seen at least every two years with the same protocol to evaluate the natural course of the disease.

The study was conducted within the Italian LCH Registry (RICLa).

Written informed consent was obtained from the parents of affected children or directly from the patients if they were older than 18 years of age.

### Data collection and statistical analysis

Demographics and data on organ involvement, treatment, and disease course were stored in a dedicated database.

The date of onset of LCH was that of either histological diagnosis or the onset of isolated DI when the latter occurred as the first clinical manifestation. The onset date of ND-LCH corresponded to the first abnormal MRI. The molecular analysis of *BRAF^V600E^* mutation was carried out through digital droplet PCR on fresh biopsy or Archived Formalin-Fixed Paraffin-Embedded (FFPE) tissue.

Continuous time variables (age at LCH onset, age at the study entry) were summarized by median and range, time elapsed between treatment prescription and its beginning was expressed by mean and standard deviation (SD), and comparisons between groups were made using Mann-Whitney test or Student’s T-Test. When appropriate confidence intervals (CI) were calculated using exact likelihood. Level of significance was set at 5% two sided.

Categorical data were reported as frequencies. The comparison between dichotomous variables was performed through the Chi Quadro Test.

## Results

### Study population

Twenty-two patients (9 females) with ND-LCH were routinely evaluated at Meyer Hospital.

Eleven of the 22 patients (50%) exhibited MRI findings of ND-LCH and at least abnormal SEPs and were enrolled for IVIG treatment. Specifically, at the time of treatment indication, 5 patients (45%) had mild abnormal MRI findings (grading=1) while 6 patients (55%) showed MRI grading > 1. MRS disclosed an abnormal NAA/Cr ratio in the cerebellum in 7 patients (64%). Neurological examination was abnormal in 10 patients (91%), 2 of whom [#7, #15] were severely symptomatic with dysphagia, dysarthria, ataxia, and bradykinesia. The SARA scale revealed an abnormal score (range, 1–39) in 8 patients, with the highest value of 39 in patient #7 and 14 in #15. Besides neurological abnormalities, Patient #2 had serious behavioral changes. However, assessing psychiatric deterioration was outside the scope of the present study.

SEPs were abnormal in all patients, as required for treatment indication. The N20 response was absent bilaterally in 2, absent on the left in 2, absent on the right in 1, delayed bilaterally in 2 and delayed on the left side in 4; the N13 response was absent bilaterally in 1.

BAEPs were abnormal in 4 patients; the I-V interval was delayed bilaterally in 2 and delayed on the left in 1, peaks I and V were abnormal in 1.

All patients with indications according to the above-mentioned criteria for the therapeutic study were treated starting in 2017, either soon (mean 2.7 months and SD 3.05) after indication [#4, #26, #41] or later (mean 39.5 months, SD 6.36) because of the initial refusal of the parents [#16, #34].

The remaining 11 patients were untreated. At first evaluation, 6 patients (54%) presented mild pathological findings at MRI (grading=1) while in the other 5, the grading was > 1 (45%). MRS disclosed an abnormal NAA/Cr ratio in the cerebellum of 4 patients (36%). SARA score of 1 was observed in 1 subjectively healthy patient (9%).

SEPs were normal in all patients (100%). BAEPs were abnormal in 2 patients (18%); the I-V interval was delayed to the right side in #17 and abnormal in #31.

Demographics and clinical data of patients with and without indications for IVIG treatment are summarized in **Table 1** and details on evaluations in **Supplementary Table 1**.

**Table 1:** Demographics and clinical data of ND-LCH patients with and without indications to IVIG treatment.

	Without indication – Not treated (11 subjects)	With indication – Treated (11 subjects)	Study population (22 subjects)
Gender, N (%)			
Female	5 (45.45)	4 (36.36)	9 (40.91)
Male	6 (54.55)	7 (63.64)	13 (59.09)
*p-value from chi^2^ test*	*0.665*		
Age at enrolment, months			
Mean (SD)	69.91 (73.22)	113.45 (83.28)	91.68 (79.70)
*p-value from t-student test*	*0.208*		
Median (Interquartile Range)	42 (28-78)	86 (69-141)	71.50 (42-108)
*p-value from Mann-Whitney test*	***0.033***		
Age at diagnosis of LCH, months			
Mean (SD)	24.09 (24.26)	30.36 (17.89)	27.23 (21.05)
*p-value from t-student test*	*0.498*		
Median (Interquartile Range)	18 (10-32)	25 (19-38)	22.50 (12-35)
*p-value from Mann-Whitney test*	*0.138*		
Age at diagnosis of ND, months			
Mean (SD)	69.64 (73.34)	74.91 (45.48)	72.27 (59.61)
*p-value from t-student test*	*0.842*		
Median (Interquartile Range)	42 (28-78)	69 (44-81)	59 (34-77)
*p-value from Mann-Whitney test*	*0.189*		
Difference between diagnosis of ND and LCH, months			
Mean (SD)	45.55 (53.08)	42.18 (44.63)	43.86 (47.89)
*p-value from t-student test*	*0.874*		
Median (Interquartile Range)	18 (13-68)	31 (10-52)	29 (13-52)
*p-value from Mann-Whitney test*	*0.974*		
Multiple sites, N (%)			
No	3 (27.27)	5 (45.45)	8 (36.36)
Yes	8 (72.73)	6 (54.55)	14 (63.64)
*p-value from chi^2^ test*	*0.375*		
Diabetes Insipidus or Craniofacial bone lesions, N (%)			
Only one risk factors	6 (54.55)	5 (45.45)	11 (50)
Both	5 (45.45)	6 (54.55)	11 (50)
*p-value from chi^2^ test*	*0.670*		
Reactivation, N (%)			
No	4 (36.36)	2 (18.18)	6 (27.27)
Yes	7 (63.64)	9 (81.82)	16 (72.73)
*p-value from chi^2^ test*	*0.338*		
BRAF V600E mutations, N (%)			
No	2(18.18)	7 (63.64)	9(40.91)
Yes	6 (54.55)	3 (27.27)	9 (40.91)
NA	*3 (27.27)*	*1 (9.09)*	4 (18.18)
*p-value from chi2 test*	*0.092*		
History of chemotherapy for ND-LCH before study entry			
No	1 (9.09)	–	1 (4.55)
Yes	10 (90.91)	11 (100.00)	21 (95.45)
*p-value from chi^2^ test*	–		
Chemotherapy for ND-LCH at time of study entry			
No	9 (81.82)	9 (81.82)	18 (81.82)
Yes	2 (18.18)	2 (18.18)	4 (18.18)
*p-value from chi^2^ test*	*1.000*		
Grading MRI >1 at first evaluation			
Yes	5 (45.45)	6 (54.45)	11 (50.00)
No	6 (54.45)	5 (45.45)	11 (50.00)
*p-value from chi^2^ test*	*1.000*		
Abnormal SARA first evaluation			
Yes	1 (9.10)	10 (90.90)	11 (50.00)
No	10 (90.90)	1 (9.10)	11 (50.00)
*p-value from chi^2^ test*	***<0.001***		
Pathological BAEPs first evaluation			
Yes	2 (18.18)	4 (36.36)	6 (27.28)
No	9 (81.82)	7 (63.64)	16 (72.72)
*p-value from chi^2^ test*	*0.632*		
Follow-up from first pathological MRI			
Mean (SD)	48.36 (16.97)	66.00 (17.26)	57,18 (18.98)
*p-value from t-student test*	0.025*		
Median (Interquartile Range)	48 (34-63)	71 (48-81)	63 (40-72)
*p-value from Mann-Whitney test*	***0.018***		
Follow-up from treatment indication			
Mean (SD)	–	53.27 (24.38)	–
Median (Interquartile Range)	–	56 (35-71)	–
Follow-up from treatment start			
Mean (SD)	–	24.55 (15.06)	–
Median (Interquartile Range)	–	20 (12-32)	–

ND, neurodegenerative; LCH, Langerhans cell Histiocytosis; MRI, magnetic resonance imaging.

Bold values indicate statistically significant differences between two groups of patients.

"-" indicates that it was not possible to apply the statistical test between the two groups.

**Figure 1:**
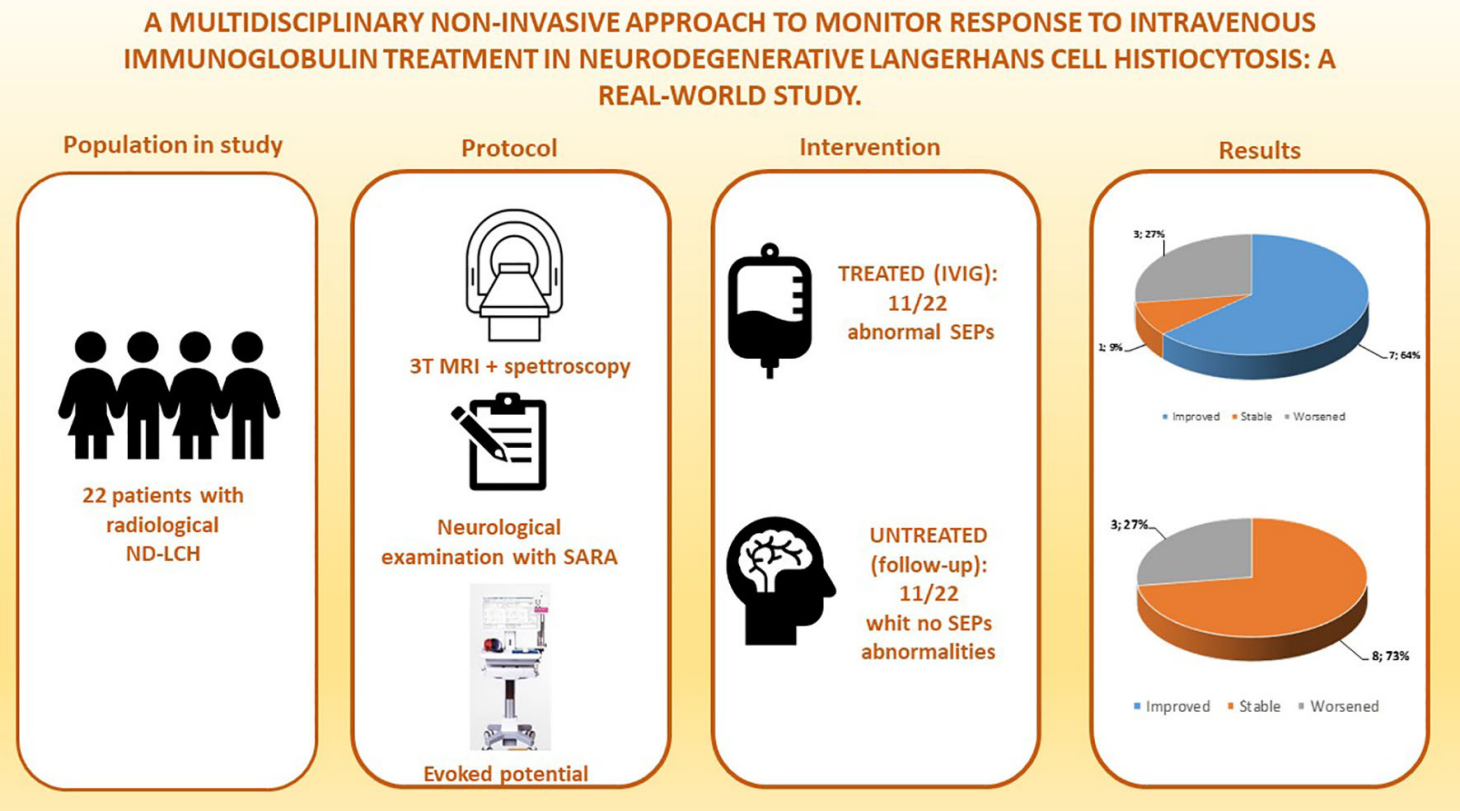
An overview of the monitoring protocol and the main results of the study.

An overview of the monitoring protocol and the main results is reported in **Figure 1**.

### Treated patients

Eleven patients (7 males, 4 females) received monthly IVIG for a median time of 1.7 years (range, 1 – 5.1 years) and were seen annually after the first pathological MRI for a median time of 5.9 years (range, 2.9 – 7.3 years).

At last evaluation, MRI remained stable in all patients (100%) while MRS worsened in 1 (9%), improved in 1 (9%) and remained stable in the remaining 9 patients (82%).

Neurological examinations improved in 7 patients (64%), reaching a complete normalization in 4 (36%), were stable in 2 (18%) and severely worsened in the remaining 2 (18%) [#7, #15]. The latter group of patients were seriously symptomatic at first evaluation and stopped IVIG treatment after 1.7 and 1.5 years, respectively.

SEPs measures improved with a complete normalization in 4/9 evaluated patients (44.5%), remained stable in 4 (44.5%) and worsened in 1 (11%) [#8] with right P14-20 delayed interval. BAEPs normalized in 2/9 evaluated patients (22%), remained stable in 6/9 (67%) and worsened in 1 (11%) [#8] with bilateral increased I-V interval. Patients #7 and #15 did not perform evoked potential because of clinical deterioration.

Thus, 3/11 (27%) worsened in at least one examination (neurological or neurophysiologic), while 7/11 (63%) improved and 1 (10%) remained stable.

Patients with or without worsening of at least one diagnostic parameter were compared in order to identify early predictors of treatment failure. Clinical and demographic characteristics of these patients are reported in **Table 2**. Patients who worsened had experienced a significantly longer time elapse between the diagnosis of LCH and the diagnosis of ND-LCH and a more severe MRI (grading=4) with respect to those who remained stable or had improved. No statistical difference was found with respect to other parameters at treatment initiation.

**Table 2:** Clinical and demographical characteristics of patients with or without worsening to at least one diagnostic parameter.

	Without Worsening (n=8)	With Worsening (n=3)
Age at diagnosis of LCH, months		
Mean (SD)	29.75 (20.48)	32.00 (11.27)
*p-value from t-student test*	*0.864*	
Median (Interquartile Range)	24 (20 – 31.50)	38 (19 - 39)
*p-value from Mann-Whitney test*	*0.414*	
Months between LCH diagnosis and ND diagnosis		
Mean (SD)	46.88 (43.85)	175.33 (104.33)
*p-value from t-student test*	***0.014***	
Median (Interquartile Range)	36.50 (11 – 71.50)	143 (91 – 292)
*p-value from Mann-Whitney test*	***0.025***	
Multiple sites, N (%)		
No	4 (50.00)	1 (33.33)
Yes	4 (50.00)	2 (66.67)
*p-value from chi^2^ test*	*0.621*	
History of chemotherapy for ND-LCH before study entry		
No	–	–
Yes	8 (100.00)	3 (100.00)
*p-value from chi^2^ test*	*-*	
Diabetes Insipidus, N (%)		
No	3 (37.50)	2 (66.67)
Yes	5 (62.50)	1 (33.33)
*p-value from chi^2^ test*	*0.387*	
Craniofacial bone lesions, N (%)		
No	–	
Yes	8 (100.00)	3 (100.00)
*p-value from chi^2^ test*	*-*	
Reactivation, N (%)		
No	2 (25.00)	–
Yes	6 (75.00)	3 (100.00)
*p-value from chi^2^ test*	*-*	
BRAF V600E mutations, N (%)		
No	6 (50.00)	2 (66.67)
Yes	2 (37.50)	1 (33.33)
NA	1 (12.50)	–
*p-value from chi^2^ test*	*-*	
Grading at 1st MRI evaluation, N (%)		
1	5 (62.50)	–
2	3 (37.50)	–
3	–	
4		3 (100.00)
*p-value from chi^2^ test*	*-*	
Mean (SD)	1.38 (0.52)	4.00 (0)
*p-value from t-student test*	***<0.001***	
Median (Interquartile Range)	1 (1 – 2)	4 (-)
*p-value from Mann-Whitney test*	***0.009***	
Spectroscopy at 1^st^ evaluation		
Normal	5 (62.50)	–
Pathological	3 (37.50)	3 (100.00)
*p-value from chi^2^ test*	*-*	
NE at 1^st^ evaluation		
Normal	2 (25.00)	1 (33.33)
Pathological	6 (75.00)	2 (66.67)
*p-value from chi^2^ test*	*0.782*	
SEPs at 1^st^ evaluation		
Normal	2 (25.00)	–
Pathological	6 (75.00)	3 (100.00)
*p-value from chi^2^ test*	*-*	
BAEPS at 1^st^ evaluation		
Normal	6 (75.00)	1 (33.33)
Pathological	2 (25.00)	2 (66.67)
*p-value from chi^2^ test*	*0.201*	

ND, neurodegenerative; LCH, Langerhans cell Histiocytosis; MRI, magnetic resonance imaging; NE, neurological examination; SEPs, Somatosensory evoked potentials; BAEPs, Brain-stem auditory evoked potentials.

Bold values indicate statistically significant differences between two groups of patients.

"-" indicates that it was not possible to apply the statistical test between the two groups.

### Patients without indication

Eleven patients (6 males, 5 females) with no treatment indication were followed-up with at least every two years for a median time of 4 years (1.8 – 6 years) from the first pathological MRI.

At last evaluation, MRI remained stable in all patients (100%). MRS worsened in 3 patients (27%), improved in 2 (18%) and remained stable in 6 (55%). Neurological examination worsened in 3 (27%) patients [#6, #17, #31] and remained stable in the remaining 8 (73%). SEPs worsened in 1 patient (9%) [#31] and remained stable in the remaining 10 (91%). BAEPs worsened in 2 patients (18%) [#17, #31] and remained stable in the remaining 9 (82%). Thus, 0/11 improved, 8/11 (73%) remained stable and 3 (27%) worsened.

At the final follow-up, patient #31, due to worsening SEPs, was indicated to treatment and started monthly IVIG.

## Discussion

We report on the follow-up of 22 patients with ND-LCH evaluated through a standardized multi-disciplinary protocol for a median of 5.2 years since their first abnormal brain MRI. Patients were selected for therapeutic intervention if they had abnormal SEPs, with all but two being subjectively healthy at first evaluation. We therefore treated patients with and without overt clinical manifestations and followed them up with a standardized approach in a real-world setting.

Early treatment has been suggested to possibly prevent ND-LCH progression towards severe disabilities. Imashuku et al. observed a beneficial effect of IVIG in patients with low Expanded Disability Status Scale EDSS scores (1.0–2.5) at treatment initiation (21). Further evidence came from the murine model of ND-LCH of Mass et al. who showed that treatment with BRAF inhibitors delayed the onset of neurological manifestations when initiated early (one versus three months of age) (9).

In this study, all but two patients (#7, #15) started the treatment when still pauci- or asymptomatic. At a median time of 1.7 years from treatment start, 73% (8/11) of patients were stable or improved according to the results of our multidisciplinary protocol, thus confirming the efficacy of an early start of treatment (6, 9, 21). The three patients who worsened had a higher MRI grading at treatment initiation and faced a longer time lapse between the diagnosis of LCH and ND-LCH. Two patients had a long history of LCH with multiple reactivations and were severely symptomatic at treatment initiation. Specifically, a girl (#7) was diagnosed with single system multifocal bone LCH at the age of 8 months and with ND-LCH at 3.3 years, and a boy (#15) was diagnosed with risk organ negative MS-LCH at 3.3 years and with ND-LCH at 6.3 years. They both experienced clinical progression during treatment precluding the continuation of the study. A third patient (#8) had *BRAF* ^V600E^ risk organ negative MS-LCH diagnosed at the age of 1.8 years old and experienced multiple reactivations. He was found to have ND-LCH at the age of 8 and was treated while still asymptomatic, despite severe MRI lesions (grading 4) because of worsening of both SEPs and BAEPs at two subsequent evaluations.

The follow-up of patients who did not receive treatment for ND-LCH confirms that if left untreated ND-LCH is a slightly progressive disease within 1.1 - 4.2 years from initial diagnosis. Treatment with IVIG led to improved results in 7 patients and stable results in 1: all of them with early stage ND-LCH at treatment start. Conversely, none of the untreated patients improved in NE, SEPs or BAEPs and three worsened, as expected based on the known natural history of the disease. In addition, the comparison between treated patients with and without worsening suggests that a more severe ND-LCH at treatment initiation and a longer exposure to LCH is associated with a failure of the IVIG therapy. These findings, cumulatively, support the efficacy of early IVIG treatment in slowing down the clinical progression of the disease and the usefulness of neurological and neurophysiological evaluation for the monitoring of ND-LCH. Brain MRI remained stable at last follow-up in both treated and untreated patients, which confirms that subtle changes are not detectable by conventional neuroimaging in the short term.

As additional findings, clinical and demographic features did not differ between patients with and without indication for treatment, thus confirming that the selection for treatment cannot be based on these features. More severe clinical findings at first evaluation in the treated group further support patient selection and treatment effectiveness.

This study has limitations. The sample size is small due to the rarity of the condition and the difficulties in recruitment. A larger study in the setting of a multicenter prospective trial is warranted. Neuropsychological evaluations were not included in our protocol because they did not show significant results in our previous study (6). Biological study were not available at our center at the start of the study. The recently proposed CSF biomarkers i.e., neurofilament light could be used to usefully complement our protocol, if validated in larger cohorts in the setting of a prospective trial.

At the time we planned this study, the treatment of choice in our center was high dose of IVIG based on the results of previous studies from the Japanese group (21, 26).

Recent evidence of perivascular infiltrations by *BRAF* ^V600E^ positive monocytes in brain biopsies of patients with ND-LCH paved the way to MAPK targeted therapies (10). McClain et al. reported initial clinical and imaging improvement in ¾ of patients with mild clinical ND-LCH treated with *BRAF^V600E^* inhibitors, while the fourth patient with long-standing disease progressed (10). However, dedicated, and well-structured clinical trials are required to confirm the efficacy of MAPK driven therapies in ND-LCH. One of the main limitations to the development of such trials is the lack of a standardized method to select patients in the early stages of the disease and to monitor their response to therapy. We think our multidisciplinary protocol could overcome these limitations.

## Conclusions

This study confirms the usefulness of a multi-disciplinary, non-invasive, low cost, protocol including a targeted MRI study, neurological examination completed by SARA and neurophysiological examination including SEPs and BAEPs, for the monitoring of therapy response and natural history of patients with ND-LCH. Our findings support the use of this multi-disciplinary protocol, combined with a neuropsychological evaluation and more invasive approaches i.e., CSF neurofilament light, in the setting of future therapeutical trials for ND-LCH.

## Data Availability

The raw data supporting the conclusions of this article will be made available by the authors, without undue reservation.
