# Supplementary material for: A multidisciplinary non-invasive approach to monitor response to intravenous immunoglobulin treatment in neurodegenerative Langerhans cell histiocytosis: a real-world study
Source: Front Immunol. 2024 Aug 16;15:1422802. doi: 10.3389/fimmu.2024.1422802 (PMC11361919; doi:10.3389/fimmu.2024.1422802)
Supplement: Supplementary file 1 [file Table1.docx]

SUPPLEMENTARY TABLE 1. Results of baseline and yearly evaluations for patient

|  | **At ND diagnosis** | **1-year evaluation** | **2-year evaluation** |  | **3-year evaluation** |  | **4-year evaluation** |  | **5-year evaluation** |  | **6-year evaluation** |  | **7-year evaluation** | |
| --- | --- | --- | --- | --- | --- | --- | --- | --- | --- | --- | --- | --- | --- | --- |
| **With indication - Treated** | | | | | | | | | | | | | |  |
| **#41** | MRI: Grading 1  NE: Pathological  SARA: 2  SEPs: Normal  BAEPS: Normal | MRI: Stable  NE: Stable  SARA: 2  SEPS: Pathological  BAEPS:Pathological  **Indication: YES**  **Treatment: YES** | MRI: Stable  NE: Improved  SARA: 0  SEPS: NA  BAEPS: Improved |  |  |  |  |  |  |  |  |  |  | |
| **#26** | MRI: Grading 1  NE: Normal  SARA : 0  SEPS: Normal  BAEPS: Normal | MRI: Stable  NE: Normal  SARA : 0  SEPS: Normal  BAEPS: Normal | MRI: Stable  NE: Pathological  SARA: 3  SEPS:Pathological BAEPS: Normal **Indication: YES**  **Treatment: YES** |  | MRI: Stable  NE: Improved  SARA: 2  SEPS: Improved  BAEPS: Normal |  |  |  |  |  |  |  |  | |
| **#15** | MRI: Grading 4  NE: Pathological  SARA: 14  SEPS: Pathological  BAEPS: Pathological  **Indication: YES** | MRI: Stable  NE: NA  SARA : 14  SEPS: Stable  BAEPS: Stable | MRI: Stable  NE: Worsened  SARA: 27  SEPS: Stable  BAEPS: Stable  **Treatment: YES** |  | MRI: Stable  NE: Worsened  SARA: 27  SEPS: NA  BAEPS: NA |  |  |  |  |  |  |  |  | |
| **#34** | MRI: Grading 1  NE: Pathological  SARA: 3  SEPS: Pathological  BAEPS: Normal  **Indication: YES** | MRI: Stable  NE: Stable  SARA : 3  SEPS: Stable  BAEPS: Normal  **Treatment: YES** | MRI: Stable  NE: Improved  SARA: 0  SEPS: Stable  BAEPS: Normal |  |  |  |  |  |  |  |  |  |  | |
| **#10** | MRI: Grading 1  NE: Normal  SARA: 0  SEPS: Pathological  BAEPS: Normal  **Indication: YES** | MRI: Stable  NE: Pathological  SARA: 1  SEPS: Stable  BAEPS: Normal | MRI: Stable  NE: Stable  SARA : 1  SEPS: Stable  BAEPS: Normal |  | MRI: Stable  NE: Stable  SARA : 1  SEPS: Stable  BAEPS: Normal |  | MRI: Stable  NE: Worsened  SARA: 2  SEPS: Stable  BAEPS: Normal |  | MRI: Stable  NE: Worsened  SARA: 3  SEPS: Stable  BAEPS: Normal  **Treatment: YES** |  | MRI: Stable  NE: Improved  SARA: 0  SEPS: Stable  BAEPS: Normal |  | MRI: Stable  NE: Stable  SARA : 0  SEPS: Stable  BAEPS:Normal | |
| **#2** | MRI: Grading 2  NE: Pathological  SARA: 1  SEPS: Pathological  BAEPS: Pathological  **Indication: YES** | MRI: Stable  NE: Worsened  SARA: 2  SEPS: Worsened  BAEPS: Stable | MRI: Stable  NE: Worsened  SARA: 3  SEPS: Stable  BAEPS: Stable **Treatment: YES** |  | MRI: Stable  NE: Stable  SARA: 3  SEPS: Stable  BAEPS: Stable |  | MRI: Stable  NE: Improved  SARA: 1  SEPS: Stable  BAEPS: Improved |  |  |  |  |  |  | |
| **#7** | MRI: Grading 4  NE: Pathological  SARA: 39  SEPS: Pathological  BAEPS: Pathological  **Indication: YES** | MRI: Stable  NE: Worsened  SARA: 39  SEPS: Stable  BAEPS: Stable  **Treatment: YES** | MRI: Stable  NE: Worsened  SARA: 39  SEPS: NA  BAEPS: NA |  |  |  |  |  |  |  |  |  |  | |
| **#16** | MRI: Grading 2  NE: Pathological  SARA: 2  SEPS: Pathological  BAEPS: Normal | MRI: Stable  NE: Stable  SARA: 2  SEPS: Stable  BAEPS: Normal  **Indication: YES** | MRI: Stable  NE: Stable  SARA: 2  SEPS: Worsened  BAEPS: NA |  | MRI: Stable  NE: Worsened  SARA: 3  SEPS: Worsened  BAEPS:Pathological  **Treatment: YES** |  | MRI: Stable  NE: Improved  SARA: 1  SEPS: Improved  BAEPS: Stable |  |  |  |  |  |  |  |
| **#8** | MRI: Grading 4  NE: Normal  SARA: 0  SEPS: Pathological  BAEPS: Normal  **Indication: YES** | MRI: Stable  NE: Normal  SARA: 0  SEPS: NA  BAEPS: Normal | MRI: Stable  NE: Normal  SARA: 0  SEPS: Worsened  BAEPS: Normal |  | MRI: Stable  NE: Normal  SARA: 0  SEPS: Stable  BAEPS: Normal  **Treatment: YES** |  | MRI: Stable  NE: Normal  SARA: 0  SEPS: Improved  BAEPS: Normal |  | MRI: Stable  NE: Normal  SARA: 0  SEPS: Stable  BAEPS: Normal |  | MRI: Stable  NE: Normal  SARA: 0  SEPS: Worsened  BAEPS:Pathological |  |  |  |
| **#1** | MRI: Grading 1  NE: Pathological  SARA: 1  SEPS: Pathological  BAEPS:Pathological  **Indication: YES** | MRI: Stable  NE: Stable  SARA: 1  SEPS: Worsened  BAEPS: Stable | MRI: Stable  NE: Stable  SARA: 1  SEPS: Stable  BAEPS: Stable  **Treatment: YES** |  | MRI: Stable  NE: Stable  SARA: 1  SEPS: Stable  BAEPS: Stable |  | MRI: Stable  NE: Stable  SARA: 1  SEPS: Stable  BAEPS: Stable |  | MRI: Stable  NE: Stable  SARA: 1  SEPS: Stable  BAEPS: Stable |  |  |  |  |  |
| **#4** | MRI: Grading 2  NE: Pathological  SARA: 1  SEPS: Pathological  BAEPS: Normal  **Indication: YES** | MRI: Stable  NE: Stable  SARA: 1  SEPS: Stable  BAEPS: Normal **Treatment: YES** | MRI: Stable  NE: Improved  SARA: 0  SEPS: Improved  BAEPS: Normal |  | MRI: Stable  NE: Stable  SARA: 0  SEPS: Improved  BAEPS: Normal |  | MRI: Stable  NE: Stable  SARA: 0  SEPS: Stable  BAEPS: Normal |  | MRI: Stable  NE: Stable  SARA: 0  SEPS: Stable  BAEPS: Normal |  | MRI: Stable  NE: Stable  SARA: 0  SEPS: Stable  BAEPS: Normal |  |  |  |
| **Without indication – No treated** | | | | | | | | | | | | | |  |
| **#9** | MRI: Grading 3  NE: Normal  SARA: 0  SEPS: Normal  BAEPS: Normal | MRI: Stable  NE: Normal  SARA: 0  SEPS: Normal  BAEPS: Normal | MRI: Stable  NE: Normal  SARA: 0  SEPS: Normal  BAEPS: Normal |  | MRI: Stable  NE: Normal  SARA: 0  SEPS: Normal  BAEPS: Normal |  |  |  |  |  |  |  |  |  |
| **#32** | MRI: Grading 3  NE: Normal  SARA: 0  SEPS: Normal  BAEPS: Normal | MRI: Stable  NE: Normal  SARA: 0  SEPS: NA  BAEPS: NA |  |  |  |  |  |  |  |  |  |  |  |  |
| **#3** | MRI: Grading 3  NE: Normal  SARA: 0  SEPS: Normal  BAEPS: Normal | MRI: Stable  NE: Normal  SARA: 0  SEPS: Normal  BAEPS: Normal | MRI: Stable  NE: Normal  SARA: 0  SEPS: Normal  BAEPS: Normal |  |  |  |  |  |  |  |  |  |  |  |
| **#11** | MRI: Grading 1  NE: Normal  SARA: 0  SEPS: Normal  BAEPS: Normal | MRI: Stable  NE: Normal  SARA: 0  SEPS: Normal  BAEPS: Normal | MRI: Stable  NE: Normal  SARA: 0  SEPS: Normal  BAEPS: Normal |  |  |  |  |  |  |  |  |  |  |  |
| **#12** | MRI: Grading 2  NE: Normal  SARA: 0  SEPS: Normal  BAEPS: Normal | MRI: Stable  NE: Normal  SARA: 0  SEPS: Normal  BAEPS: Normal |  |  |  |  |  |  |  |  |  |  |  |  |
| **#13** | MRI: Grading 1  NE: Normal  SARA: 0  SEPS: Normal  BAEPS: Normal | MRI: Stable  NE: Stable  SARA: 0  SEPS: NA  BAEPS: NA | MRI: Stable  NE: NA  SARA: NA  SEPS: Normal  BAEPS: Normal |  | MRI: Stable  NE: Normal  SARA: 0  SEPS: Normal  BAEPS: Normal |  |  |  |  |  |  |  |  |  |
| **#35** | MRI: Grading 1  NE: Normal  SARA: 0  SEPS: Normal  BAEPS: Normal | MRI: Stable  NE: Normal  SARA: 0  SEPS: Normal  BAEPS: Normal | MRI: Stable  NE: Normal  SARA: 0  SEPS: Normal  BAEPS: Normal |  |  |  |  |  |  |  |  |  |  |  |
| **#6** | MRI: Grading 1  NE: Normal  SARA: 0  SEPS: Normal  BAEPS: Normal | MRI: Stable  NE: Pathological  SARA: 1  SEPS: Normal  BAEPS: Normal | MRI: Stable  NE: Worsened  SARA: 2  SEPS: Normal BAEPS: Normal |  |  |  |  |  |  |  |  |  |  |  |
| **#48** | MRI: Grading 1  NE: Pathological  SARA: 1  SEPS: Normal  BAEPS: Normal | MRI: Stable  NE: NA  SARA: 1  SEPS: Normal  BAEPS: Normal | MRI: Stable  NE: Stable  SARA: 1  SEPS: Normal  BAEPS: Normal |  |  |  |  |  |  |  |  |  |  |  |
| **#17** | MRI: Grading 1  NE: Normal  SARA: 0  SEPS: Normal  BAEPS: Pathological | MRI: Stable  NE: Normal  SARA: 0  SEPS: Normal  BAEPS: Worsened | MRI: Stable  NE: Pathological  SARA: 7  SEPS: Normal  BAEPS: Stable |  | MRI: Stable  NE: Stable  SARA: 7  SEPS: Normal  BAEPS: Stable |  |  |  |  |  |  |  |  |  |
| **#31** | MRI: Grading 2  NE: Normal  SARA: 0  SEPS: Normal  BAEPS: Pathological | MRI: Stable  NE: Pathological  SARA: 1  SEPS: Worsened  BAEPS:Pathological  **Indication: YES** |  |  |  |  |  |  |  |  |  |  |  |  |

Legend: MRI=Magnetic resonance imaging, NE= Neurological examination; SEPs= Somatosensory evoked potentials; BAEPs= Brain-stem auditory evoked potentials, NA=not avalable. The grey shaded areas represent patients undergoing IVIG treatment
